# Creatine Kinase as a Prognostic Factor for Mortality in Extracorporeal Cardiopulmonary Resuscitation: A Retrospective Observational Study

**DOI:** 10.3390/jcm14238404

**Published:** 2025-11-27

**Authors:** Dong Ki Kim, Byeong Jo Chun, Yeon Ji Seong

**Affiliations:** 1 Department of Emergency Medicine, Chonnam National University Hospital, Gwangju 61469, Republic of Korea; cnuem00068@jnu.ac.kr (D.K.K.); duswl5831@naver.com (Y.J.S.); 2 Department of Emergency Medicine, Chonnam National University Medical School, Gwangju 61469, Republic of Korea

**Keywords:** extracorporeal cardiopulmonary resuscitation, creatine kinase, biomarker

## Abstract

**Background/Objectives**: Extracorporeal cardiopulmonary resuscitation (ECPR) is increasingly used for refractory cardiac arrest, yet overall survival and neurologic recovery remain poor. We examined whether creatine kinase (CK) levels measured at predefined times after ECPR were associated with in-hospital mortality and evaluated their discriminatory performance. **Methods**: We retrospectively analyzed adults (≥18 years) who underwent ECPR at a single tertiary center between January 2015 and December 2022. CK was measured at 4, 12, 24, and 48 h after ECPR initiation; lactate at 24 and 48 h. The primary outcome was in-hospital mortality. For each CK time point, we built multivariable logistic regression models adjusted for age, sex, initial rhythm, and total arrest time. Discrimination was assessed using receiver operating characteristic curves. **Results**: Of 183 patients screened, 102 met the inclusion criteria; 29 (28.4%) survived to discharge. Median total arrest time was longer in non-survivors than in survivors, 40.0 min (28.0–58.0) vs. 30.0 min (20.0–44.0; *p* = 0.008). CK at 4 h showed limited discrimination (area under the curve = 0.567). CK at 24 and 48 h was higher in non-survivors (24 h: 5444 vs. 3954 U/L, *p* = 0.045; 48 h: 4793 vs. 2234 U/L, *p* = 0.003), with the highest predictive value at 48 h (area under the curve = 0.69; optimal cutoff = 2001 U/L). **Conclusions**: CK levels after ECPR were independently associated with in-hospital mortality, with the moderate predictive performance at 48 h after initiation. CK may serve as an adjunct to risk assessment and management in patients undergoing ECPR.

## 1. Introduction

Extracorporeal cardiopulmonary resuscitation (ECPR) use in cardiac arrest is increasing. Evidence supports its use in selected patients, such as those with refractory cardiac arrest unresponsive to conventional CPR (cCPR) or refractory ventricular fibrillation [1]. Based on these data, major resuscitation guidelines recommend selective ECPR [2]. Nonetheless, survival after ECPR is approximately 28% [3], and only about 21.3% of patients are discharged with favorable neurologic outcomes [4]. Improving outcomes requires not only appropriate patient selection but also reliable indicators that reflect patient condition during treatment and inform decisions [4]. Such indicators, which capture ongoing physiology during ECMO, may serve as treatment-phase markers to inform real-time management [3].

Recent discussions regarding the variable effects of adrenaline during resuscitation—including reports of outcome divergence during the pandemic—have further highlighted the need for reliable, physiology-based indicators that reflect patient status during ECPR [5]. Such indicators may complement established parameters and support more individualized treatment-phase assessment.

During ECPR and ECMO, femoral cannulation, altered limb perfusion, systemic low-flow states, and vasoconstriction can precipitate ischemic myopathy and rhabdomyolysis [6,7], which are closely linked to organ dysfunction, particularly acute kidney injury (AKI), and contribute to adverse outcomes [7]. Creatine kinase (CK) is a representative marker of skeletal and myocardial injury [8]. In critically ill patients, elevated CK levels have been associated with greater use of renal replacement therapy (RRT), longer hospitalization, and higher mortality [9], suggesting potential clinical value in ECPR.

No single biomarker can fully capture the heterogeneous physiological changes that occur after ECPR. Identifying additional indicators that reflect injury types not recognized by conventional markers may allow more precise detection of clinical deterioration [10]. While lactate is widely used to represent the degree of global hypoperfusion [11], CK provides complementary information by reflecting the burden of skeletal and myocardial injury that frequently accompanies ECPR and ECMO. Incorporating this tissue-injury dimension into bedside assessment may enhance the utility of existing biomarkers and support a more comprehensive evaluation of patient status following cannulation [6,8].

We hypothesized that CK would predict in-hospital mortality after ECPR. We therefore analyzed CK trajectories after ECPR to assess associations with mortality and their potential utility for status assessment, prognostication, and treatment decisions.

## 2. Materials and Methods

### 2.1. Study Design and Setting

This single-center, retrospective observational study was conducted at Chonnam National University Hospital. Patients underwent ECPR according to standardized institutional criteria and treatment protocols between January 2015 and December 2022.

The main indications were:

(a) Refractory cardiac arrest unresponsive to cCPR;
(b) Cardiac or cardiopulmonary etiologies considered potentially reversible (e.g., acute myocardial infarction, decompensated heart failure, pulmonary embolism);
(c) Anticipated arrest-to-ECMO-flow time ≤ 60 min (i.e., low-flow ≤ 60 min);
(d) Signs of life during ongoing CPR (e.g., spontaneous limb movement or gasping) suggesting possible return of spontaneous circulation.

At our institution, ECPR is performed primarily for patients with suspected cardiac etiology of arrest, in accordance with institutional protocol. ECPR may also be considered for non-cardiac causes (e.g., toxicologic or other reversible conditions) if recovery is deemed reasonably likely. This study was approved by the Institutional Review Board of the Biomedical Research Institute at Chonnam National University Hospital (CNUH-2025-295). Given its retrospective design using de-identified records, the requirement for informed consent was waived. Demographic, clinical, and laboratory data were extracted from the electronic medical record.

### 2.2. Study Population and Eligibility Criteria

We included adult patients (≥18 years) who underwent ECPR.

Inclusion criteria:

(a) ECPR performed by clinicians at our hospital;
(b) Use of veno-arterial (VA) ECMO for ECPR.

Exclusion criteria:

(a) Known malignancy;
(b) Died immediately after ECPR, precluding acquisition of clinical or laboratory data;
(c) Cardiac arrest primarily due to systemic inflammatory or infectious etiologies (e.g., hypoxia from pneumonia, sepsis) or clearly non-cardiac in origin;
(d) Immediate surgical intervention required or ECPR performed for intraoperative cardiac arrest;
(e) Absence of any CK measurement. (At our hospital, CK testing is not mandatory for ECMO patients and is performed at the treating physician’s discretion.)

**Figure 1:**
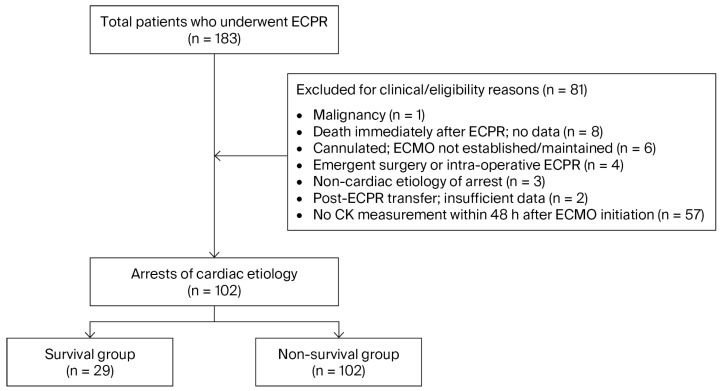
Patient enrollment and analysis flow. Of 183 patients who underwent ECPR between January 2015 and December 2022, 24 were excluded for clinical or eligibility reasons, and 57 were excluded for lack of CK measurement within 48 h after ECMO initiation, yielding a final analytic cohort of 102 patients.

From January 2015 to December 2022, 183 patients underwent ECPR at Chonnam National University Hospital. We excluded 1 patient with known malignancy; 8 who died immediately after ECPR with unavailable clinical and laboratory data; 6 in whom ECMO cannulation was achieved but support could not be established or maintained (e.g., cardiac tamponade); 4 requiring immediate surgery (e.g., acute aortic dissection) or intraoperative ECPR; 3 with non-cardiac arrest etiologies (e.g., sepsis-related hypoxia); and 2 transferred after ECPR initiation elsewhere with insufficient initial data or no follow-up information. Additionally, 57 patients lacking CK measurements within 48 h of ECMO initiation were excluded because CK testing was not protocol-mandated at our institution. The final analytic cohort comprised 102 patients (Figure 1). After exclusions, all remaining cases were of cardiac-origin arrest.

### 2.3. Data Collection

We collected data on demographics, comorbidities, cardiac-arrest features, treatment variables, and laboratory measurements.

Demographics and comorbidities included age, sex, hypertension, diabetes mellitus, dyslipidemia, cerebrovascular disease, chronic kidney disease, and cardiovascular history (percutaneous coronary intervention, myocardial infarction, heart failure, angina, prior cardiac arrest). Arrest characteristics included out-of-hospital cardiac arrest status, witnessed arrest, initial cardiac rhythm (shockable vs. non-shockable), bystander CPR (basic life support), ECPR time, defined as the arrest-to-ECMO-flow interval, and total arrest time, defined as the sum of no-flow and low-flow time. Treatment variables included targeted temperature management (TTM) and continuous RRT (CRRT). Laboratory measurements obtained immediately after ECPR initiation included platelet count, blood urea nitrogen, creatinine, total bilirubin, and lactate. Additionally, CK and lactate were measured repeatedly at regular intervals after ECPR. CK values were collected at 4, 12, 24, and 48 h after ECPR initiation, and lactate values at 24 and 48 h. Only patients with at least one CK measurement were included in the analysis; handling of subsequent missing values is described in Section 2.4.

The primary clinical outcome was in-hospital mortality.

### 2.4. Statistical Analysis

Baseline characteristics were summarized and compared between survivors and non-survivors. Continuous variables were assessed for normality and are reported as mean ± standard deviation or median (interquartile range [IQR]). Categorical variables are reported as frequency and percentage. Between-group comparisons used the Student’s *t*-test or Wilcoxon rank-sum test for continuous variables and the chi-square test or Fisher’s exact test for categorical variables.

We performed multivariable logistic regression to assess the independent association between CK and in-hospital mortality. A forced-entry approach with prespecified covariates (age, sex, initial shockable rhythm, and total arrest time) was applied. To avoid multicollinearity among highly correlated CK measurements, separate models were fitted for each time point (4, 12, 24, and 48 h). CK values were log10-transformed to reduce skewness, and adjusted odds ratios (ORs) with 95% confidence intervals (CIs) were reported. The events-per-variable ratio was approximately 14–15, supporting model stability.

Changes in CK levels over time were evaluated descriptively at prespecified intervals (4, 12, 24, and 48 h). Between-group differences (survivors vs. non-survivors) were assessed using two-sided Wilcoxon rank-sum tests for continuous variables and Fisher’s exact test for categorical variables (e.g., CPC). A formal within-subject longitudinal analysis was not undertaken because sampling intervals and available measurements were non-uniform in this retrospective dataset.

We further evaluated the discriminatory performance of CK at each time point using receiver operating characteristic (ROC) analyses, calculated the area under the curve (AUC) with 95% CIs via the DeLong method, and identified the optimal cut-off values using the Youden index.

We addressed missing data using multiple imputation by chained equations with a random forest algorithm to generate five complete datasets. The first imputed dataset was used for descriptive statistics and visualizations, and pooled estimates from all five datasets, per Rubin’s rules, were used for regression and ROC analyses.

Two-sided significance was set at *p* < 0.05. All analyses were conducted in R (version 4.3 or later).

## 3. Results

### 3.1. Baseline Characteristics

Of 102 patients, 29 (28.4%) survived to hospital discharge. Most demographic features, comorbidities, and arrest characteristics were similar between groups. However, total arrest time was longer in non-survivors than in survivors (median [IQR] 40.0 [28.0–58.0] vs. 30.0 [20.0–44.0] min; *p* = 0.008).

CRRT was more frequent in non-survivors than in survivors (67.1% vs. 31.0%; *p* < 0.001), whereas TTM use did not differ significantly (*p* = 0.089).

In initial post-ECPR laboratory results, creatinine was higher in non-survivors than in survivors (median [IQR] 1.5 [1.2–1.8] vs. 1.3 [1.0–1.5] mg/dL; *p* = 0.010). Lactate measured immediately after ECPR was also higher in non-survivors (15.0 (13.0–18.3) mmol/L) than in survivors (13.1 (11.6–14.8) mmol/L; *p* = 0.013)

Survivors experienced markedly longer stays in both the intensive care unit and the hospital compared with non-survivors. Median ICU stay was 8.9 days (IQR 6.2–19.5) for survivors versus 3.9 days (IQR 1.1–10.9) for non-survivors (*p* < 0.001). Similarly, the median hospital stay was 21.1 days (IQR 15.6–53.6) among survivors compared with 4.9 days (IQR 1.5–13.6) among non-survivors (*p* < 0.001). Neurologic status at discharge also differed significantly between the two groups (*p* < 0.001). (Table 1).

### 3.2. Comparison of CK Levels by Outcome

**Figure 2:**
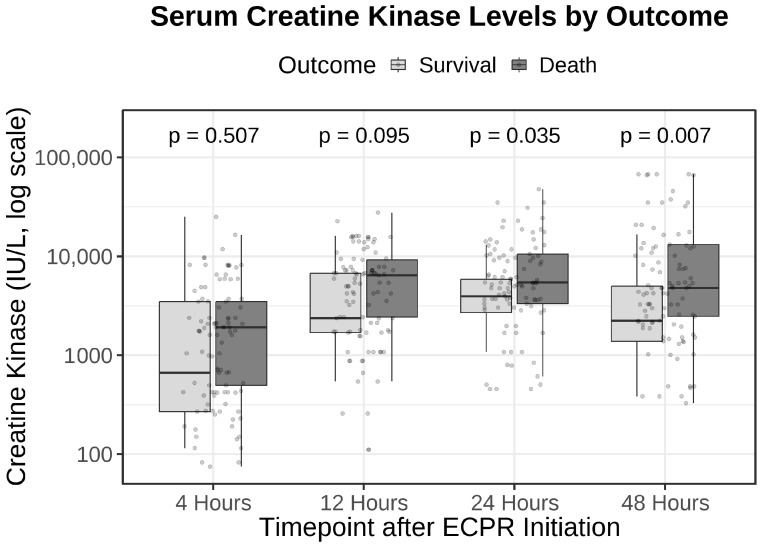
Serum creatine kinase (CK) levels by outcome after ECPR. Box-and-whisker plots display log10-transformed CK concentrations at 4, 12, 24, and 48 h after ECPR initiation, stratified by outcome (survival vs. death). Between-group comparisons at each time point were performed using the two-sided Wilcoxon rank-sum test (4 h: *p* = 0.507; 12 h: *p* = 0.095; 24 h: *p* = 0.035; 48 h: *p* = 0.007). Boxes indicate interquartile ranges with median lines; whiskers denote the 10th–90th percentiles; and points represent observations outside this range. Numbers at risk (survivors/non-survivors) at each time point are shown below the x-axis. Plots are based on the first imputed dataset for visualization; regression and ROC estimates were obtained from pooled imputations per Rubin’s rules.

Serial CK values at 24 h and 48 h were significantly higher in non-survivors than in survivors (24 h: median [IQR], 5444 [3315–10,575] vs. 3954 [2696–5874] IU/L; *p* = 0.035; 48 h: 4793 [2482–13,150] vs. 2234 [1384–5006] IU/L; *p* = 0.007). Lactate at 24 h and 48 h likewise was higher in non-survivors than in survivors (24 h: 5.5 [2.7–11.0] vs. 1.8 [1.4–2.8] mmol/L; 48 h: 2.9 [1.7–7.2] vs. 1.2 [1.0–1.9] mmol/L; both *p* < 0.001; see Table 1). Figure 2 shows the temporal distribution of CK levels by outcome; groups began to diverge after 12 h, with significantly higher CK concentrations in non-survivors at 24 h and 48 h.

### 3.3. ROC Analysis

ROC analysis evaluated the predictive performance of CK for in-hospital mortality at each post-ECPR time point. At 4 h, discrimination was limited (AUC = 0.567; optimal cutoff, 430 IU/L; sensitivity, 75.6%; specificity, 40%). Predictive ability improved over time and peaked at 48 h (AUC = 0.644; optimal cutoff, 3262 IU/L; sensitivity, 60.5%; specificity, 60%; see Table 2).

### 3.4. Multivariable Logistic Regression Analysis

In multivariable models adjusting for age, sex, initial rhythm, and total arrest time, higher CK levels were independently associated with in-hospital mortality at later time points (12, 24, and 48 h). In Model 1 (CK at 12 h), CK was a significant predictor of mortality (adjusted OR, 3.63; 95% CI, 1.01–13.09; *p* = 0.048). In Model 2 (CK at 24 h), the association was stronger (adjusted OR, 8.18; 95% CI, 1.62–41.28; *p* = 0.012). In Model 3 (CK at 48 h), the association was strongest (adjusted OR, 20.49; 95% CI, 2.72–154.05; *p* = 0.005) (Table 3).

## 4. Discussion

The principal finding is that CK was significantly associated with in-hospital mortality after ECPR, with discrimination strengthening over time and peaking at 48 h.

During ECPR and VA-ECMO, large-bore femoral cannulation and reduced limb perfusion can cause distal ischemia. Together with systemic low flow and vasoconstriction, these factors promote ischemic myopathy and rhabdomyolysis [7]. In this context, greater skeletal muscle and focal ischemic injury are linked to worse outcomes, as rhabdomyolysis is a major cause of acute kidney injury (AKI), a frequent ECMO complication. AKI is a key prognostic indicator associated with increased mortality and resource use [12,13]. Mechanistically, myonecrosis releases myoglobin and intracellular solutes, provoking tubular toxicity, oxidative stress, pigment cast formation, intrarenal vasoconstriction, and systemic complications such as hyperkalemia, metabolic acidosis, compartment syndrome, and disseminated intravascular coagulation [14,15]. Each can destabilize patients on ECMO and worsen prognosis. Given this pathophysiology, CK can serve as an indicator of injury burden in patients undergoing ECPR and VA-ECMO.

Prior studies also suggest that elevated CK may be associated with prognosis in patients receiving ECMO after cardiac arrest, including those treated with ECPR. Chen et al. reported that, among adults treated with ECMO after cardiac arrest, day 3 CK was significantly associated with successful weaning and survival, implying that CK reflects ongoing ischemic or muscular injury and recovery potential [16]. Similarly, a pediatric ECPR study demonstrated a significant association between day 3 CK and survival [17], and another pediatric ECMO cohort found higher CK values in non-survivors than in survivors, although not statistically significant [18].

Other ECPR studies link CK with markers of poor prognosis, although not always directly with outcomes. In one ECPR cohort, patients with AKI had significantly higher CK than those without AKI [19], suggesting that elevated CK accompanies complications that worsen clinical outcomes. Rilinger et al. observed that ECPR patients with lower initial pulse pressure had significantly higher CK concentrations, and lower pulse pressure was independently associated with poor prognosis [20]. In a multicenter analysis comparing cCPR with mechanical CPR, CK levels were significantly higher in the cCPR group, which also had poorer outcomes [21]. Collectively, these findings indicate that CK elevation reflects the extent of tissue and ischemic injury during ECPR and ECMO and is consistently associated with worse clinical outcomes.

Prior adult and pediatric ECMO/ECPR studies have demonstrated that higher or persistently elevated CK values are associated with AKI, unsuccessful weaning, and mortality, supporting our findings that CK reflects ischemic and muscle-injury burden. These studies collectively reinforce the physiologic plausibility of CK as an adjunct biomarker distinct from lactate-based assessments.

In our study, CK trajectories differed between survivors and non-survivors. CK peaked at 12 h in non-survivors and at 24 h in survivors; although both subsequently declined, concentrations at 24 h and 48 h remained significantly higher in non-survivors. This delayed decline and sustained elevation suggest persistent muscle and ischemic injury, indicating more severe and prolonged tissue damage in patients with poor outcomes. Comparable patterns have been reported previously. In an ECPR cohort, CK measured at 72 h was significantly lower in survivors and successfully weaned patients than in non-survivors [16]. In patients who developed limb ischemia during ECMO, CK decreased more slowly after 72–96 h, reflecting delayed recovery from ischemic injury [22]. Similarly, in pediatric rhabdomyolysis, CK normalization was delayed in patients who developed AKI (7 days) compared with those without renal involvement (6 days), although the difference was not statistically significant [23]. Together, these observations support that higher and persistently elevated CK, with delayed return to baseline, are associated with poor prognosis after ECPR.

Lactate was measured at 24 h and 48 h in most patients, but it was not included in the primary regression models because its strong global predictive power could overshadow or attenuate the independent information provided by CK. Lactate is already recognized as a well-established marker of systemic hypoperfusion and has consistently shown high discrimination for mortality in ECPR cohorts [24]. Including such a dominant variable would make it statistically difficult to assess whether CK—reflecting skeletal-muscle and limb ischemic burden—adds clinically distinct prognostic value. Therefore, we reported lactate descriptively and focused the multivariable analysis on CK timing, aiming to explore whether CK can serve as an additional, phenotype-specific biomarker rather than a substitute for lactate.

Despite these limitations, the study maintained strong internal validity through strict eligibility criteria and provided a systematic, time-resolved analysis of CK as a prognostic indicator. We found that CK concentration was associated with in-hospital mortality in ECPR patients, with the greatest predictive performance at 48 h. In practice, 48-h CK may be used as an adjunct to risk assessment after ECPR. Patients with persistently elevated CK could prompt closer surveillance for limb ischemia or rhabdomyolysis-related AKI and earlier renal support, whereas declining CK may suggest recovery of ischemic burden. These results suggest that CK may serve as a useful biomarker for risk stratification and for guiding therapeutic strategies in ECPR. Given its moderate discrimination, CK should be integrated with other clinical and laboratory indicators rather than used in isolation. Future studies are needed to validate these findings and to define the optimal timing and decision thresholds for CK measurement.

## 5. Limitations

This study had limitations. First, it was a retrospective analysis, and CK determination was not protocol-mandated; thus, some patients lacked CK measurements and were excluded. This exclusion reflected operational practice rather than outcomes and was necessary because CK was the primary exposure. Although survivor-selection bias cannot be entirely excluded, our objective was to examine the association between contemporaneously available CK values and in-hospital mortality, and we prespecified the analytic cohort accordingly. Because this was a retrospective dataset with non-uniform sampling intervals, CK values were compared between groups at each time point rather than through a formal within-subject longitudinal analysis. Second, data on procedures or complications beyond CRRT and TTM were not available. However, during the study period, no cases of compartment syndrome requiring fasciotomy occurred in the cannulated limb. Third, CK-MB was not routinely measured. Because our objective was to quantify the overall skeletal-muscle and limb ischemic burden that develops during ECPR, total CK was selected a priori as the more appropriate marker. CK-MB, being myocardial-specific, is less informative in this context, where injury is predominantly driven by rhabdomyolysis. Fourth, our study included only patients with cardiac-origin arrest, as non-cardiac cases were excluded during data cleaning. This focus is consistent with current international recommendations, which primarily endorse ECPR for potentially reversible cardiac etiologies. Therefore, the restriction does not substantially limit the applicability of our findings. Finally, the relatively small sample size and single-center design limit generalizability.

## 6. Conclusions

CK measured after ECPR was independently associated with in-hospital mortality, with moderate discriminatory performance that peaked at 48 h. CK is best used as an adjunct, adding a tissue-injury dimension to treatment-phase assessment, rather than as a standalone prognostic test.

## Figures and Tables

**Table 1:** Baseline Characteristics of Patients Undergoing ECPR.

Characteristic	Overall (*n* = 102)	Survival (*n* = 29)	Non-Survival (*n* = 73)	*p*-Value
Demographics				
Age, years	62.7 [53.1, 71.0]	61.0 [51.1, 67.1]	64.1 [54.0, 72.0]	0.059
Male, *n* (%)	87 (85.3)	24 (82.8)	63 (86.3)	0.800
Comorbidities, *n* (%)				
Hypertension	50 (49.0)	17 (58.6)	33 (45.2)	0.200
Diabetes mellitus	45 (44.1)	13 (44.8)	32 (43.8)	>0.999
Dyslipidemia	15 (14.7)	6 (20.7)	9 (12.3)	0.400
Cerebrovascular accident	10 (9.8)	2 (6.9)	8 (11.0)	0.700
Chronic kidney disease	7 (6.9)	2 (6.9)	5 (6.8)	>0.999
Cardiac history				
Prior PCI	28 (27.5)	8 (27.6)	20 (27.4)	>0.999
Myocardial infarction	23 (22.5)	6 (20.7)	17 (23.3)	0.800
Heart failure	5 (4.9)	1 (3.4)	4 (5.5)	>0.999
Angina	16 (15.7)	6 (20.7)	10 (13.7)	0.400
Arrest characteristics, *n* (%)				
Out-of-hospital cardiac arrest	23 (22.5)	5 (17.2)	18 (24.7)	0.400
Witnessed arrest	98 (96.1)	28 (96.6)	70 (95.9)	>0.999
Bystander BLS	97 (95.1)	27 (93.1)	70 (95.9)	0.600
Initial shockable rhythm	35 (34.3)	12 (41.4)	23 (31.5)	0.300
ECPR and arrest details				
ECPR time, min	30.0 [21.0, 40.0]	28.0 [20.0, 34.0]	30.0 [22.0, 44.0]	0.120
Total arrest time, min	38.0 [25.0, 53.0]	30.0 [20.0, 44.0]	40.0 [28.0, 58.0]	0.008
Treatments, *n* (%)				
TTM	30 (29.4)	5 (17.2)	25 (34.2)	0.089
CRRT	58 (56.9)	9 (31.0)	49 (67.1)	<0.001
Initial laboratory findings				
Platelets, ×10^3^/μL	139 [99.0, 185]	138 [103, 193]	140 [99.0, 174]	0.600
BUN, mg/dL	22.3 [16.7, 30.0]	20.3 [15.6, 26.4]	22.5 [17.5, 32.0]	0.200
Creatinine, mg/dL	1.5 [1.1, 1.8]	1.3 [1.0, 1.5]	1.5 [1.2, 1.8]	0.010
Total bilirubin, mg/dL	0.8 [0.5, 1.2]	0.7 [0.5, 1.1]	0.8 [0.5, 1.2]	0.600
Lactate at initiation, mmol/L	14.7 [12.3, 16.3]	13.1 [11.6, 14.8]	15.0 [13.0, 18.3]	0.013
Serial laboratory values				
CK at 4 h, IU/L	1737 [423, 3499]	666 [270, 3499]	1922 [497, 3499]	0.140
CK at 12 h, IU/L	5341 [1726, 7807]	2380 [1699, 6761]	6438 [2427, 9219]	0.067
CK at 24 h, IU/L	5272 [3315, 10,091]	3954 [2696, 5874]	5444 [3315, 10,575]	0.035
CK at 48 h, IU/L	4307 [2046, 12,075]	2234 [1384, 5006]	4793 [2482, 13,150]	0.007
Peak CK, IU/L	5255 [1922, 11,845]	3671 [1802, 6163]	6336 [2206, 12,369]	0.089
Lactate at 24 h, mmol/L	3.7 [1.9, 8.1]	1.8 [1.4, 2.8]	5.5 [2.7, 11.0]	<0.001
Lactate at 48 h, mmol/L	2.2 [1.2, 6.6]	1.2 [1.0, 1.9]	2.9 [1.7, 7.2]	<0.001
Outcomes				
ICU Duration, days	6.5 [1.9, 14.3]	8.9 [6.2, 19.5]	3.9 [1.1, 10.9]	<0.001
Hospital Duration, days	10.0 [2.5, 24.4]	21.1 [15.6, 53.6]	4.9 [1.5, 13.6]	<0.001
CPC at Discharge, *n* (%)				<0.001
1	14 (13.7)	14 (48.3)	0 (0.0)	
2	7 (6.9)	7 (24.1)	0 (0.0)	
3	5 (4.9)	5 (17.2)	0 (0.0)	
4	3 (2.9)	3 (10.3)	0 (0.0)	
5	73 (71.6)	0 (0.0)	73 (100.0)	

Data are presented as median [interquartile range] or *n* (%). Abbreviations: PCI, percutaneous coronary intervention; BLS, basic life support; ECPR, extracorporeal cardiopulmonary resuscitation; TTM, targeted temperature management; CRRT, continuous renal replacement therapy; BUN, blood urea nitrogen; CK, creatine kinase.

**Table 2:** ROC Analysis of Creatine Kinase for Predicting In-Hospital Mortality.

Variable	AUC (95% CI)	Optimal Cutoff (IU/L)	Sensitivity	Specificity
CK at 4 h	0.567 (0.422–0.712)	430	0.756	0.4
CK at 12 h	0.619 (0.422–0.815)	2404	0.751	0.538
CK at 24 h	0.630 (0.506–0.754)	5909	0.447	0.779
CK at 48 h	0.644 (0.498–0.791)	3262	0.605	0.6

Abbreviations: ROC, receiver operating characteristic; AUC, area under the curve; CI, confidence interval; CK, creatine kinase.

**Table 3:** Multivariable Logistic Regression Analysis of Predictors of In-Hospital Mortality.

Predictor Variable	Adjusted OR (95% CI)	*p*-Value
Model 1 (CK at 12 h)		
Age (per year)	1.09 (1.03–1.16)	0.004
Total arrest time (per min)	1.06 (1.02–1.09)	0.002
Male (vs. female)	0.79 (0.17–3.61)	0.764
Shockable rhythm (yes vs. no)	0.51 (0.16–1.61)	0.249
CK at 12 h (log10-transformed)	3.63 (1.01–13.09)	0.048
Model 2 (CK at 24 h)		
Age (per year)	1.11 (1.04–1.19)	0.003
Total arrest time (per min)	1.05 (1.02–1.09)	0.003
Male (vs. female)	0.81 (0.16–4.05)	0.794
Shockable rhythm (yes vs. no)	0.46 (0.14–1.50)	0.197
CK at 24 h (log10-transformed)	8.18 (1.62–41.28)	0.012
Model 3 (CK at 48 h)		
Age (per year)	1.14 (1.04–1.25)	0.007
Total arrest time (per min)	1.06 (1.02–1.11)	0.008
Male (vs. female)	0.66 (0.10–4.42)	0.657
Shockable rhythm (yes vs. no)	0.25 (0.06–1.08)	0.064
CK at 48 h (log10-transformed)	20.49 (2.72–154.05)	0.005

All models were adjusted for the listed variables. *p*-values < 0.05 were considered significant. Abbreviations: OR, odds ratio; CI, confidence interval; CK, creatine kinase.

## Data Availability

The data presented in this study are available from the corresponding author upon reasonable request. The data are not publicly available due to institutional restrictions and patient confidentiality.

## Abbreviations

The following abbreviations are used in this manuscript:

AKI	Acute kidney injury
AUC	Area under the curve
BLS	Basic life support
BUN	Blood urea nitrogen
CK	Creatine kinase
CIs	Confidence interval
CRRT	Continuous renal replacement therapy
cCPR	Conventional cardiopulmonary resuscitation
ECMO	Extracorporeal membrane oxygenation
ECPR	Extracorporeal cardiopulmonary resuscitation
IABP	Intra-aortic balloon pump
IQR	Interquartile range
OR	Odds ratio
PCI	Percutaneous coronary intervention
ROC	Receiver operating characteristic
RRT	Renal replacement therapy
TTM	Targeted temperature management
VA-ECMO	Veno-arterial extracorporeal membrane oxygenation
